# Re: Basadonna LM, Passarelli F, Graps G, et al. The Influence of Music on Perceived Pain and Levels of Anxiety While Undergoing Transperineal Prostate Biopsy: The VIVALDI Trial. Eur Urol Open Sci 2026;87:107–114

**DOI:** 10.1016/j.euros.2026.06.012

**Published:** 2026-07-09

**Authors:** Ahmet Murat Bayraktar, Bilgi İşler

**Affiliations:** Department of Urology, Konya City Hospital, University of Health Sciences, Konya, Turkey

Dear Editor,

We read with great interest the randomized VIVALDI trial by Basadonna and colleagues on music listening during transperineal prostate biopsy [1]. We congratulate the authors on providing the first randomized evidence on this question in transperineal prostate biopsy. The findings prompted us to reflect on the same issue in our own practice, and we would value the authors’ perspective on several points.

The divergence between the self-reported outcomes and the objective physiological parameters gave us the greatest pause. Although visual analog scale (VAS) pain scores, distress, and satisfaction all improved significantly in the +MUSIC group, heart rate and systolic blood pressure differed little between groups (eg, postprocedural heart rate of 72 vs 71 beats/min) [1]. This is noteworthy because a measurable anxiolytic effect might be expected to produce at least a concordant directional trend in these autonomic indicators. Indeed, in the study by Chang et al [2], cited by the authors as a key reference, music significantly lowered postprocedural heart rate (*p* = 0.014) and systolic blood pressure (*p* = 0.011) during transrectal biopsy, and the European Association of Urology systematic review reported similar effects [3]. We would be interested in how the authors interpret the absence of a concordant objective effect in their cohort, and how the lack of patient and operator blinding may have shaped the observed benefit.

Second, we consider music emerging as an independent predictor of pain (Table 3) an important finding. We were also struck by the lower need for additional sedation in the +MUSIC group (37% vs 57%) [1]. Since the decision to administer additional midazolam rested with an unblinded operator who was simultaneously exposed to the music, we wonder to what extent this reflects a true reduction in patients’ need for sedation rather than variation in the operator’s threshold for administering it. The authors’ thoughts on how to disentangle these possibilities would be most welcome.

Third, we wondered about the choice of a linear regression model for the multivariable analysis, given that nonparametric tests were used for the between-group comparisons and the ordinal nature of both the VAS pain scores and the 1–5 satisfaction ratings. We would appreciate the authors’ rationale for this approach, particularly for the satisfaction outcome, and clarification as to whether the distributional assumptions underlying the linear models were examined.

Finally, the between-group pain difference (median, 1.5 points) lies close to the commonly reported minimal clinically important difference for the VAS [4], which the authors candidly describe as modest; we appreciate this forthright assessment.

None of these points diminishes the pioneering value of VIVALDI. On the contrary, they suggest opportunities for future work: a three-arm design (music, neutral auditory stimulus, standard care), with headphone delivery to prevent operator exposure and a blinded outcome assessor, would be an important next step. We thank the authors for their important contribution.

  ***Conflicts of interest:*** The authors have nothing to disclose.

  ***Declaration of Generative AI and AI-assisted technologies in the writing process:*** During the preparation of this work the authors used generative AI technologies to improve readability and language structure. After using this tool/service, the authors reviewed and edited the content as needed and take full responsibility for the content of the publication.
